# Correction to “Right sided posterior diaphragmatic hernia with liver incarceration: A case report”

**DOI:** 10.1002/ccr3.9372

**Published:** 2024-08-27

Alexi B, Sacha L, François J, Benoit M. Right sided posterior diaphragmatic hernia with liver incarceration: a case report. *Clinical Case Reports*. 2024;12(8):e9296. doi:10.1002/ccr3.9296

In the article entitled, “Right sided posterior diaphragmatic hernia with liver incarceration: A case report”, the authors' first and last names were mistakenly reversed. The authors names should read as Alexi Boitsios, Sacha Lacroix, Francois Jehaes, and Benoit Monami.

Author names have been reversed. The current list “Boitsios Alexi, Lacroix Sacha, Jehaes François, Monami Benoit” should read “Alexi Boitsios, Sacha Lacroix, François Jehaes, Benoit Monami.” Therefore, the article should be cited as “Boitsios A, Lacroix S, Jehaes F, Monami B. Right sided posterior diaphragmatic hernia with liver incarceration: A case report. *Clinical Case Reports*. 2024;12(8):e9296. doi:10.1002/ccr3.9296.”

We apologize for this error.

